# PlaTiF: A pioneering dataset for orthopedic insights in AI-powered diagnosis of tibial plateau fractures

**DOI:** 10.1038/s41597-026-06560-5

**Published:** 2026-01-07

**Authors:** Ali Kazemi, Kaveh Same, Abolfazl Zamanirad, Soodabeh Esfandiary, Ebrahim Najafzadeh, Alireza Ahmadian, Parastoo Farnia, Mohammad Hossein Nabian

**Affiliations:** 1 Medical Physics and Biomedical Engineering Department, Faculty of Medicine, Tehran University of Medical Sciences, Tehran, Iran; 2 Research Center of Biomedical Technology and Robotics (RCBTR), Imam Khomeini Hospital Complex, Tehran University of Medical Sciences, Tehran, Iran; 3 Center for Orthopedic Trans-Disciplinary Applied Research, Tehran University of Medical Sciences, Tehran, Iran; 4 Research Center for Intelligent Technologies in Medicine (RCITM), Advanced Medical Technologies and Equipment Institute (AMTEI), Tehran University of Medical Sciences, Tehran, Iran; 5 Finetech in Medicine Research Center, School of Medicine, Iran University of Medical Sciences, Tehran, Iran; 6 Medical Physics Department, School of Medicine, Iran University of Medical Sciences, Tehran, Iran

**Keywords:** Bone imaging, Biomedical engineering, Bone, Machine learning, Data publication and archiving

## Abstract

Tibial plateau fractures account for approximately 1% of skeletal fractures, with treatment strategies varying based on fracture type, displacement, and articular involvement. Diagnosis is labor-intensive, time-consuming, repetitive, and subject to considerable inter-observer variability. Automated and precise approaches could improve accuracy and efficiency in fracture severity classification. With advances in artificial intelligence (AI), especially deep learning, such techniques are increasingly applied in medicine, yet their performance depends on high-quality training data. Here, we present a first-of-its-kind open-access dataset for AI-based analysis of tibial plateau fractures. The dataset comprises 421 heterogeneous anterior-posterior radiographs from 186 patients (mean age 45.88 ± 17.54 years; 37 females, 149 males), including normal and fractured knees. Fractures were classified by expert orthopedic surgeons and radiologists using the Schatzker system: type I (14.51%), II (18.27%), III (6.45%), IV (5.91%), V (6.45%), VI (17.20%), and normal (31.18%). All images were segmented to generate tibial bone masks, supporting morphological analysis, AI training, and automated fracture assessment. This dataset facilitates AI-driven fracture detection, classification, preoperative planning, and orthopedic assistant education.

## Background & Summary

**Fig. 1:**
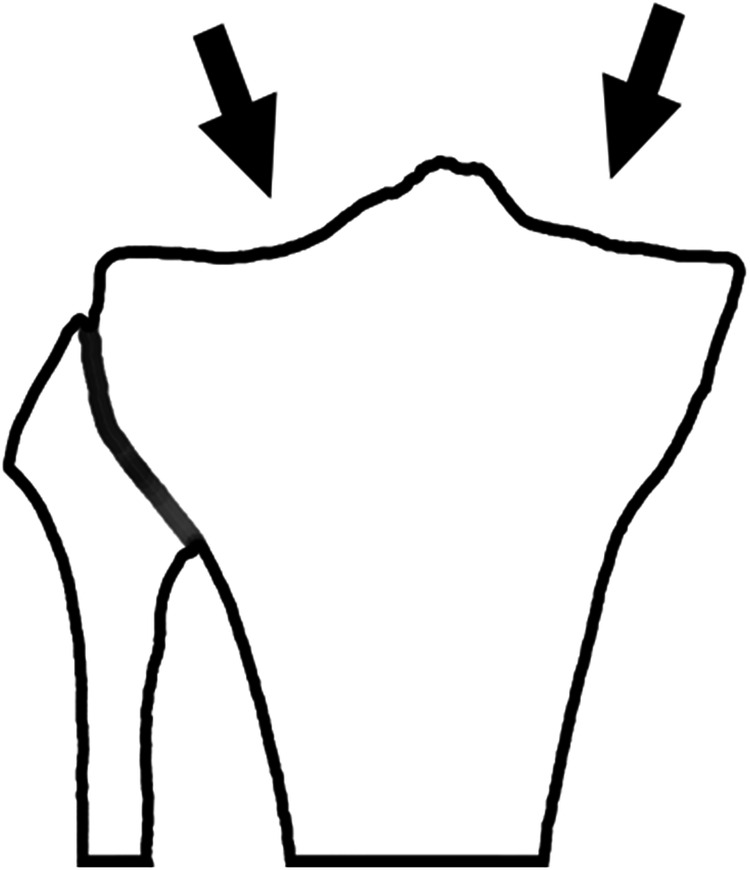
Schematic representation of the plateau surface at the proximal end of the tibia bone. Arrows point to the medial and lateral condyles.

The tibial plateau (TP) is the bony segment located at the distal part of the knee’s articular surface. The intercondylar eminence separates the TP from a concave medial condyle and a convex lateral condyle (see Fig. 1).

Fractures are frequently encountered in trauma patients, and X-ray images are the primary method used for diagnosing them^1,2^. TP fractures account for approximately 1% of all skeletal fractures while comprising nearly 8% of all fractures in patients over 55 years old^3^. In addition to affecting the bone, TP fractures may injure the surrounding soft tissue, thereby complicating treatment. A depression fracture of more than 10 mm is particularly suspected to be accompanied by cruciate ligament tears or injuries to the meniscus^4^. Furthermore, the nerves and vasculature passing posterior to the knee, particularly the sciatic nerve and the popliteal artery and vein, may be at risk of injury. The presence of such critical structures in the TP and their essential function in knee joint kinematics necessitate precise classification of fracture types, leading to accurate diagnosis, effective surgical planning, and ultimately, positive patient outcomes. Fracture classification is widely acknowledged as fundamental to fracture management and surgical intervention. It organizes knowledge, facilitates information transfer, strategizes and guides treatment, prognosticates outcomes, and enhances educational opportunities for learners. The classification and treatment of TP fractures can vary significantly based on the specific characteristics of the fracture, ranging from conservative non-surgical approaches to surgical and reconstructive procedures^5,6^.

**Fig. 2:**
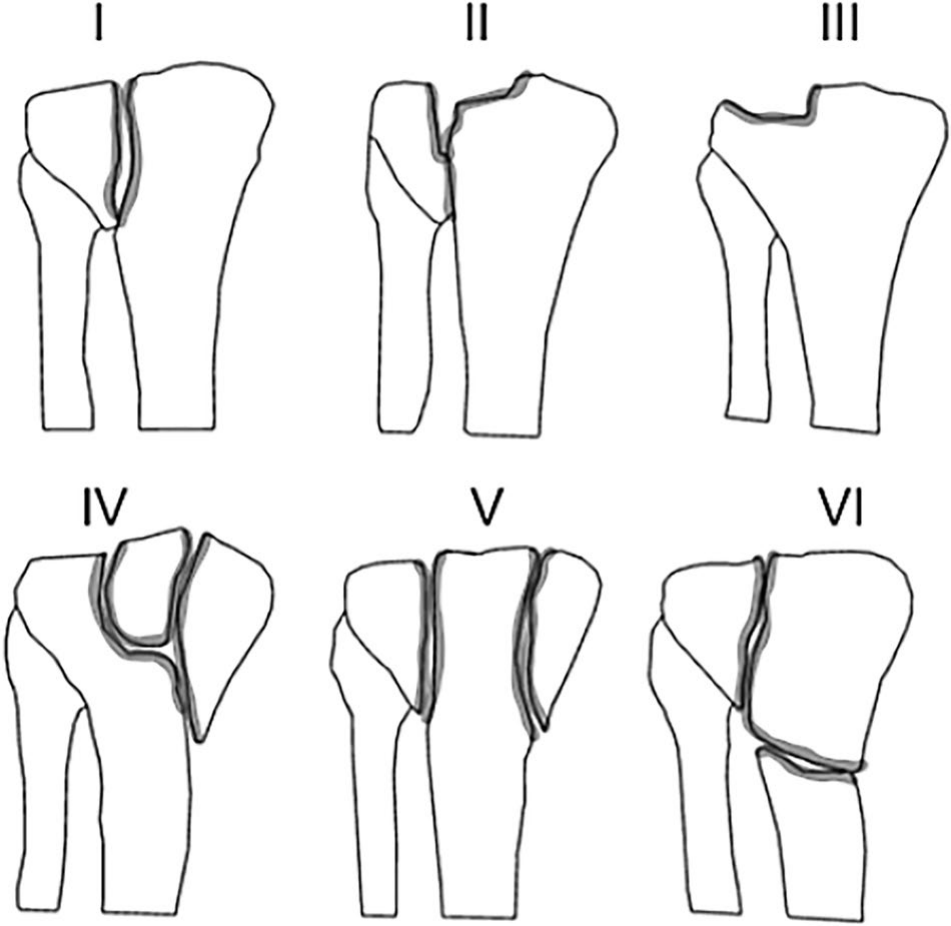
Schatzker classification of the tibial plateau fractures. Fracture types are categorized into six different classes based on the shape and severity of the fracture. Type I: Lateral plateau, split fracture, Type II: Lateral plateau, split depression fracture or fracture with articular depression, Type III: lateral plateau, depression fracture (pure depression pattern), Type IV: Internal plateau fracture (internal condyle fracture), Type V: Fracture of the bicondylar plateau (fracture of the bicondylar tibial plateau), and Type VI: Plateau fracture with separation of the metaphysis from the diaphysis.

Fracture type determination is commonly performed using the Schatzker classification system (Fig. 2), which categorizes fractures into six distinct types. Schatzker fracture type II is more frequently associated with soft tissue injuries^7^. While classification can be achieved using plain, non-weight-bearing radiographs of the knee in the anteroposterior (AP) view^8^, artifacts can represent considerable challenges for accurate classification, particularly in individuals with osteoporosis. Therefore, a CT scan of the joint is strongly recommended before any surgical planning^7,8^. On the other hand, the use of CT scans presents multiple challenges, including high costs, significant radiation exposure, sensitivity to image artifacts, and the potential for incidental findings that may lead to unnecessary interventions^9,10^. Despite its limitations, such as physicians’ uncertainty in diagnosing fracture edges, X-ray radiography has remained the primary method for diagnosing fractures for decades, owing to its low radiation dose, patient comfort, and good image quality^1,11^. As with many similar situations, the application of artificial intelligence (AI), particularly deep learning algorithms, could be beneficial in improving the diagnosis procedure and supporting radiologists and orthopedic surgeons. Deep learning algorithms have the potential to be transformative in such scenarios, as they can assist with fracture classification using only plain radiographs. A significant barrier to the development and deployment of such tools is the limited availability of high-quality training data. Accurately labeled radiographs are essential for achieving optimal outcomes. Although publicly accessible databases of knee radiographs exist, the absence of accurate labeling for TP fractures limits their utility for research and clinical applications. Despite previous studies, such as Liu *et al*.^12^, which have attempted to diagnose TP fractures using an AI model, their databases are not publicly accessible.

In addition to these challenges, segmentation of X-ray bone images is crucial for diagnosing orthopedic conditions. However, challenges arise due to high noise levels, low image contrast, overlapping anatomical structures, and variations in imaging positions, making segmentation of the tibia and surrounding bones a complex task. Numerous general medical image segmentation techniques have been adapted to address this challenge^13,14^. Moreover, the Schatzker classification system, which is used to guide clinical evaluation, treatment planning, and outcome prediction for patients with tibial plateau fractures (TPFs), is, like any diagnostic process, susceptible to human error, potentially leading to misdiagnosis and compromised prognosis, highlighting the imperative need for automated segmentation as a complementary tool to enhance diagnostic accuracy and clinical decision-making^15–17^.

This study introduces, for the first time, a publicly available and expertly annotated imaging dataset mainly focused on tibial plateau articular surface fractures, thereby addressing a significant gap in orthopedic AI resources. The key contributions are outlined as follows:

- First-of-its-Kind Public Dataset on Tibial Plateau Fractures
  We introduce the first publicly accessible imaging dataset specifically focused on tibial plateau fractures. This unique collection integrates anteroposterior (AP) X-ray radiographs and coronal CT sections of the tibia bone. Its multi-modal structure enables a comprehensive understanding of fracture morphology, making it a valuable resource for orthopedic AI research and beyond.
- Clinician-Validated Fracture Type Annotation utilizing the Schatzker Classification System
  Board-certified orthopedic surgeons precisely categorized all fractures in the dataset according to the Schatzker classification system. This expert-level annotation supports the development of robust deep learning algorithms for automated fracture diagnosis and classification while also facilitating precise pre-operative surgical planning, particularly for procedures like Open Reduction and Internal Fixation (ORIF).
- Tibial Bone Segmentation with Expert-Reviewed Boundary Annotations

A specialized annotation framework was established to ensure accurate, standardized, and reproducible delineation of tibial bone boundaries, providing high-precision binary masks. This framework significantly enhances the dataset’s utility for applications such as segmentation, anatomical modeling, and AI-driven image analysis.

Furthermore, this dataset can catalyze a variety of research directions, such as:

- Development of automated fracture classification and segmentation models using convolutional neural networks (CNNs) or transformer-based models
- Comparative studies between AI-generated predictions and clinical assessments
- Generation of synthetic data using GANs to augment fracture cases in underrepresented classes
- Foundation for future longitudinal tracking and pre-operative surgical planning based on fracture morphology and classification

## Methods

### Study design and data collection

A total of 319 patient records with a TP fracture diagnostic label were reviewed at Shariati Hospital under the supervision of Tehran University of Medical Sciences. These files corresponded to patients admitted to the hospital from June 2016 to June 2024. We extracted images according to codes related to a standardized system for diseases and medical conditions, referred to as ICD-10-CM. We searched for fracture codes related to the tibia or tibial shaft, condyles, distal end, plateau, and those involving the ankle or malleolus.

- Inclusion criteria:
  This study included patients with suspected TP fractures who underwent radiographic assessment.
- Exclusion criteria:
- Exclusion criteria comprised X-ray evidence of previous fractures, chronic deformities, bone lesions, open growth plates, specific diseases such as Osgood-Schlatter disease, avulsion fractures involving the posterior cruciate ligament (PCL) and the lateral collateral ligament (LCL), as well as a history of previous surgical interventions

Following the initial assessment and removal of records with incomplete data, 186 patients were considered for inclusion in the study. Demographic information and the duration of hospital stay for each case were also documented during the data collection process. Descriptive statistics provide information on the distribution of different fracture types; additional details are available in the dataset. All calculations were performed using Microsoft Excel 2019 for data organization and MATLAB software R2022b for the segmentation workflow. This dataset is part of a multiphase study that has been approved by the Medical Ethics Committee of Tehran University of Medical Sciences under the code IR.TUMS.MEDICINE.REC.1400.1031, and all procedures were conducted in accordance with the Declaration of Helsinki and relevant institutional guidelines. Patients presenting with suspected tibial plateau fractures at Shariati Hospital had pre-existing clinical imaging data retrospectively identified from the institutional imaging archive. Only anonymized images and demographic information were used to construct the dataset. As a result, the IRB waived the requirement for written informed consent. Also, for all data used for external validation, strict confidentiality measures were implemented: all personal identifiers (including name, national identification number, and any directly identifying patient characteristics) were removed and are not reported in any form. No additional imaging, radiation exposure, financial cost, or interference with the patients’ diagnostic or therapeutic pathway was introduced as part of this study.

### Demographics, Imaging Protocol, and Annotation

AP radiographs were available for each included sample, and diagnoses and fracture types were validated using CT scans. Fracture type diagnosis and classification were initially performed by a senior orthopedic resident, followed by a comprehensive review by an attending orthopedic surgeon. Furthermore, in cases of discrepancy, a final evaluation was conducted by an additional expert orthopedic knee surgeon. The study included 186 patients with a mean age of 45.88 years (standard deviation: 17.54), consisting of 37 females and 149 males. The distribution of fracture types according to the Schatzker classification was as follows: type I (14.51%), type II (18.27%), type III (6.45%), type IV (5.91%), type V (6.45%), and type VI (17.20%). In addition, 31.18% of patients exhibited a normal tibial plateau, with or without the presence of additional fractures. Imaging protocol to ensure consistency in data acquisition, all anteroposterior (AP) X-ray radiographs were obtained using a single Fujifilm FDR Smart Digital X-ray system. The imaging protocol adhered to standard clinical guidelines for knee radiography. Acquisition parameters were maintained within a tube voltage range of 60–80 kVp and a tube current range of 100–200 mAs. Specific exposure settings within these ranges were adjusted based on individual patient size and anatomy to ensure optimal image quality and appropriate tissue penetration. This approach reflects real-world clinical variations while ensuring protocol standardization. Table 1 provides a detailed overview of the data.

**Table 1:** Demographic data and Schatzker classification diagnosis.

Characteristics							Data
No. of patients							186
No. of X-ray Images							421
Gender:							
No. of Females^a^							37 (19.89%)
No. of Males^a^							149 (80.1%)
Age^b^							45.88 ± 17.54
Schatzker Classification Grading^c^	Type I	Type II	Type III	Type IV	Type V	Type VI	No Tibial Plateau Fracture
No. (% of total data)	27 (14.51%)	34 (18.27%)	12 (6.45%)	11 (5.91%)	12 (6.45%)	32 (17.20%)	58 (31.18%)
Imaging Protocol Details	Fujifilm FDR Smart Digital X-ray system with a voltage of 60–80 kVp and current of 100–200 mAs based on patient size and anatomy						

^a^Information provided as the number of patients (percentage).

^b^Information provided as mean ± standard deviation.

^c^Information provided as a count and percentage.

### Image segmentation workflow

**Fig. 3:**
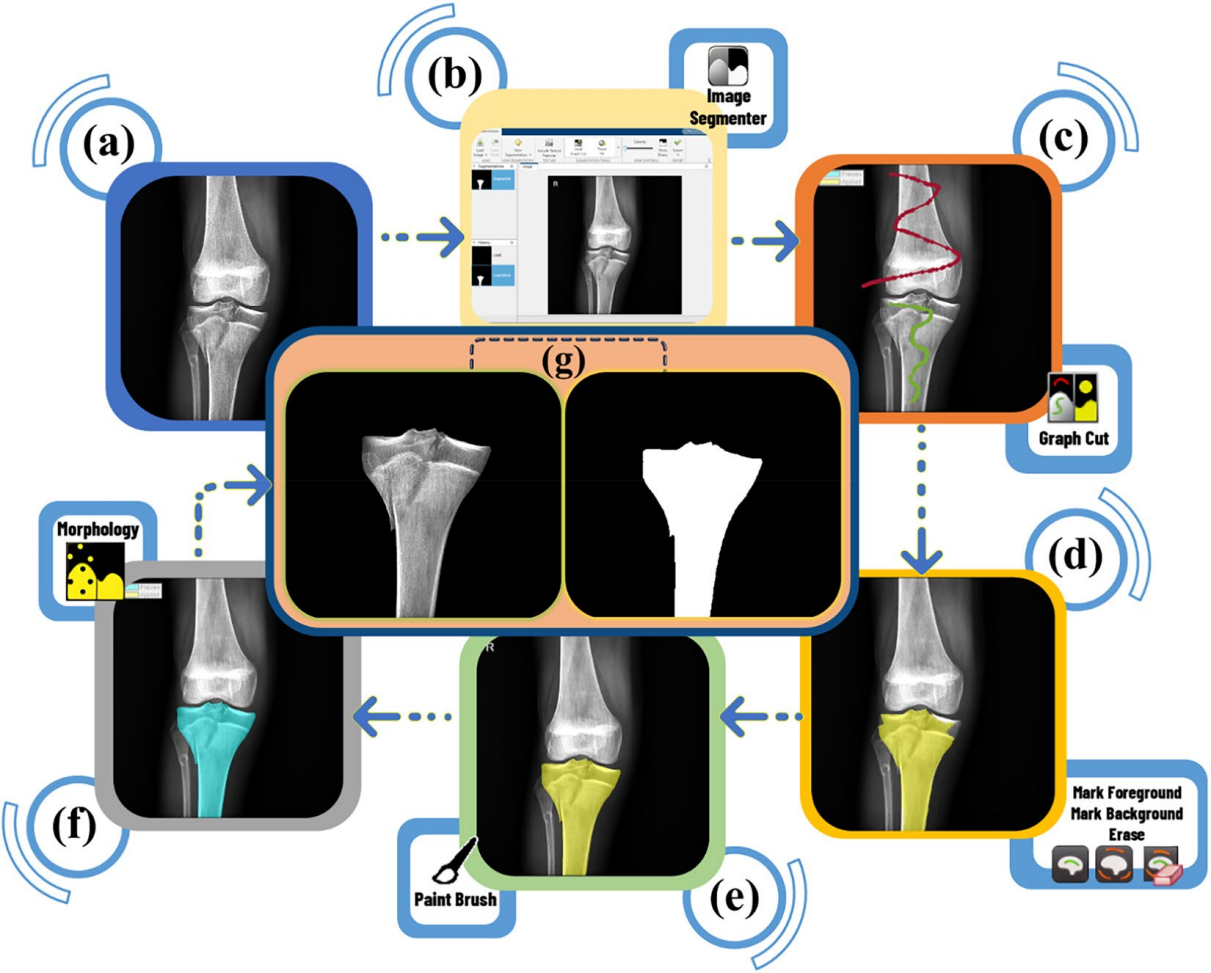
The steps of tibia segmentation in the MATLAB Image Segmenter app. (**a**) AP knee radiography, (**b**) Importing image to Image Segmenter app, (**c**) Applying Graph Cut, (**d**) Primary mask from the segmented portion after specifying foreground and background of the image (the foreground is the area we wish to segment, while the background is everything else), (**e**) New mask after applying the paint-brush tool to modify the edges of the mask and fulfill Tibia, (**f**) Final mask, in morphology tab, we apply certain operations on the mask, such as dilation and erosion, by opening and closing on the mask pixels, (**g**) Export masked image and binary mask of X-ray image to the MATLAB workspace.

Subsequently, the radiographs were manually segmented using the Image Segmenter app in the Image Processing and Computer Vision toolbox of MATLAB R2022b software. The steps of bone tibia segmentation are shown in Fig. 3.

The procedure involves the following steps:

- **Image Import**(Fig. 3a,b): AP radiographs were imported into the Image Segmenter app (Fig. 3a,b).
- **Initial Segmentation** (Fig. 3c,d): The Graph Cut algorithm is applied to create an initial segmentation. This separates the foreground, the tibia (the bone targeted for segmentation), from the background (the remaining anatomical structures in the image). As a result, an initial mask is generated for the tibial bone.
- **Mask Refinement**(Fig. 3e): The paint-brush tool is employed to refine the mask, ensuring accurate delineation of the entire tibia region (Fig. 3e). This enables manual refinement of the mask boundaries.
- **Morphological Processing (**Fig. 3f): The final step involves applying morphological operations (Fig. 3f) within the Morphology tab. These operations, including Opening and Closing, further refine the mask by eliminating minor imperfections, resulting in more accurate segmentation of the tibia region.
- **Export**(Fig. 3g): Upon achieving satisfaction with the segmentation, the masked image and the binary mask can be exported back to the MATLAB workspace for further analysis.

**Fig. 4:**
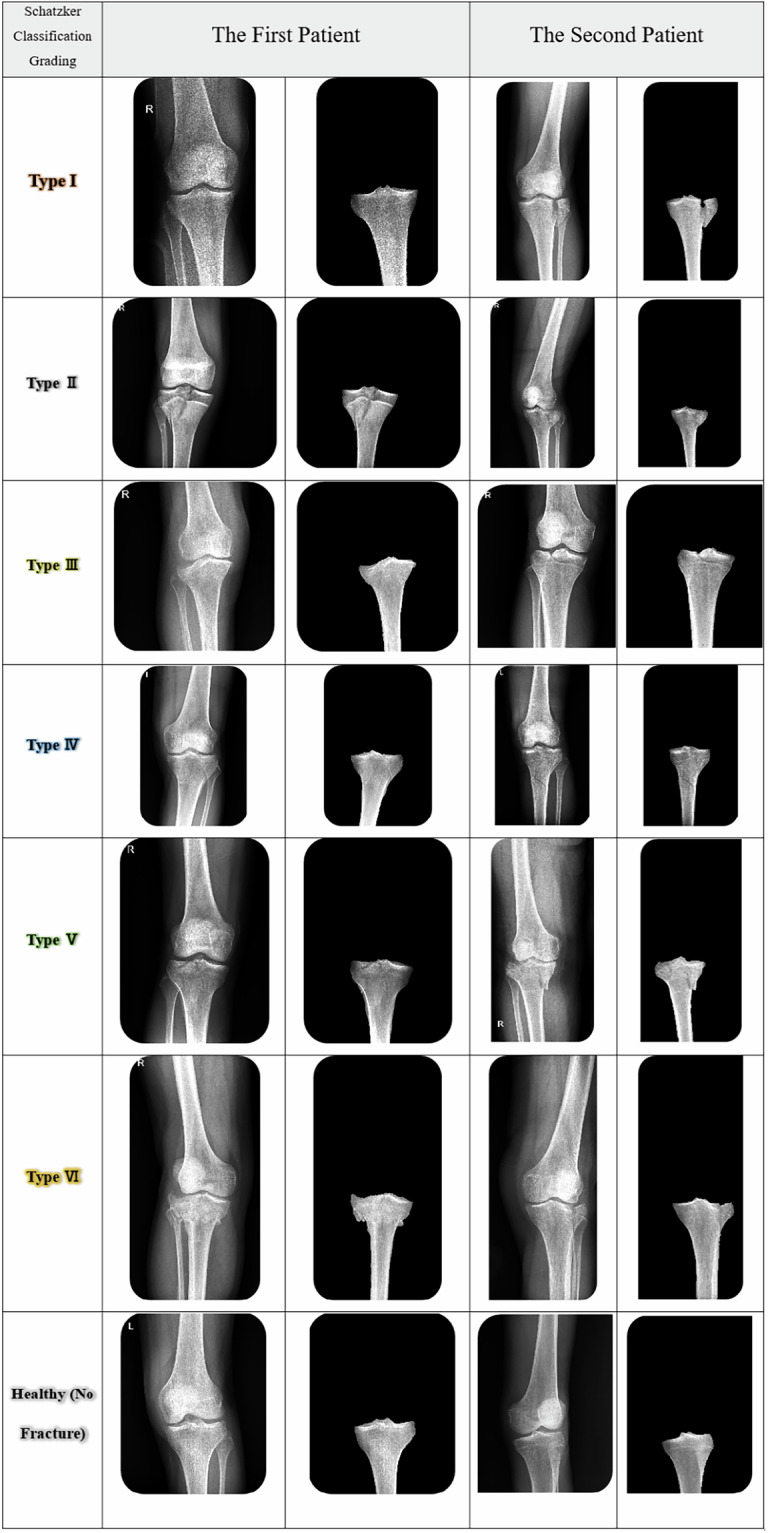
AP radiographic images of knee fractures categorized by the Schatzker classification system with tibia bone segmentation labels.

Figure 4 displays a series of radiographs illustrating various Schatzker classification patterns of knee fractures identified in a select group of patients. Additionally, the corresponding segmentation labels for the tibia bone are provided, facilitating the visualization of the anatomically relevant structures.

## Data Records

The PlaTiF dataset is permanently archived and publicly accessible at the Zenodo repository^18^ and comprises a comprehensive, open-access collection of tibial plateau fracture data from 186 patients. Each case is meticulously labeled and validated through multi-expert review processes, ensuring a high level of annotation accuracy. The dataset also includes corresponding demographic information and segmentation masks, thereby enhancing its applicability for both clinical and computational studies. As a result, this dataset holds considerable value and clinical utility across various applications. To facilitate the use of the dataset, its structure and key components are discussed in detail. The dataset includes multiple file types, the specifics of which are outlined as follows:

1. Patient Information (.xlsx file):

- Patient ID (unique identifier) to access image information
- Age (years), Gender (male/female), and Nationality
- Date of Admission and Release
- Title of Diagnosis
- Fracture Type Diagnosis Based on Schatzker Classification
- Tibial Fracture in the Right (R) or Left (L) Foot

2. Radiographic Views (.mat file):

- AP View:
  - Image file (.mat file)

Within the .mat file, each row is systematically aligned with the details of an X-ray image for each patient, specified by the patient ID in the table. This includes the original image, the binary mask of the tibia bone, the segmented tibia bone image, and the corresponding Schatzker classification annotation. Additionally, to enable a detailed assessment of intra-articular involvement, fragment depression, and posterior column fractures, a coronal CT section was acquired for each patient.

3. Quality Control Measures:

- Image preprocessing techniques are employed to enhance contrast by adjusting the image data to utilize the full dynamic range.

4. Data Availability Statement
  The dataset utilized in this study is publicly available on the Zenodo repository under the Creative Commons Attribution 4.0 International (CC BY 4.0) license and can be accessed at the following link: 10.5281/zenodo.18007397^18^.

## Technical Validation

The technical quality, accuracy, and reliability of this dataset were ensured through a multi-stage process involving standardized data curation, a rigorous annotation protocol, and a multi-expert consensus-based review. Each radiograph and its corresponding segmentation mask were independently annotated and validated by a multidisciplinary team of experts, including two orthopedic surgeons (15 and 10 years of clinical experience), an orthopedic resident (5 years of experience), and a radiologist and physicist (18 years of experience), to ensure annotation accuracy and consistency. The tibial plateau regions were manually segmented, and fracture type labels were assigned according to the Schatzker classification system, with all annotations cross-validated among reviewers to minimize inter-observer variability. The combination of expert-verified labels, manual segmentation masks, and structured metadata ensures a high level of technical quality, making the dataset suitable for computational analysis, AI model development, and benchmarking purposes. A rigorous, quality control protocol was implemented to validate all annotations and minimize inter-observer variability. Each radiograph, along with its corresponding segmentation mask and Schatzker label, was independently reviewed by at least two panel experts. Reviewers were blinded to the initial annotations and the assessments of other experts. They were tasked with either accepting the annotation or flagging it for revision, providing comments where necessary. In all cases where a disagreement occurred between reviewers (regarding either the segmentation boundary or the fracture classification), a consensus meeting was held to discuss differing interpretations and reach a final, unanimous decision. This iterative process ensures that ambiguous or complex cases receive the highest level of scrutiny, resulting in a robust ground truth. This protocol was critical to establishing the dataset’s high level of annotation accuracy.

While this dataset provides a valuable resource, its technical scope has known limitations. The dataset currently includes only anterior-posterior (AP) radiographs and lacks lateral views and computed tomography (CT) scans. In comparison, the Schatzker classification was initially based on 2D imaging; understanding the precise three-dimensional spatial location of fracture components, particularly the integrity of the rim and the configuration of wedge fragments, plays a central role in evaluating fracture stability and optimizing surgical fixation strategies. Recent advancements, as described in the work by Mauricio Kfuri and Joseph Schatzker^5,7^, have introduced an extended CT-based classification framework that incorporates detailed three-dimensional anatomical descriptors (e.g., anterior/posterior and medial/lateral modifiers). These modifications provide a more comprehensive understanding of fracture morphology and play a critical role in preoperative planning and fixation strategies. Accordingly, the absence of volumetric imaging data in this dataset may limit its applicability for studies requiring full 3D fracture characterization or posterior plane identification. However, this dataset is part of an ongoing effort, and future releases aim to include lateral radiographic views and CT images accompanied by expert-reviewed labels. This future expansion will align the dataset with current orthopedic classification standards, enhancing its utility for deep learning models in surgical decision support, 3D modeling, and fracture localization tasks. These future initiatives can lead to more innovative clinical decision support tools, improved diagnostic accuracy, enhanced preoperative planning and surgical decision-making, and personalized treatment strategies in orthopedic care.

## Usage Notes

**Fig. 5:**
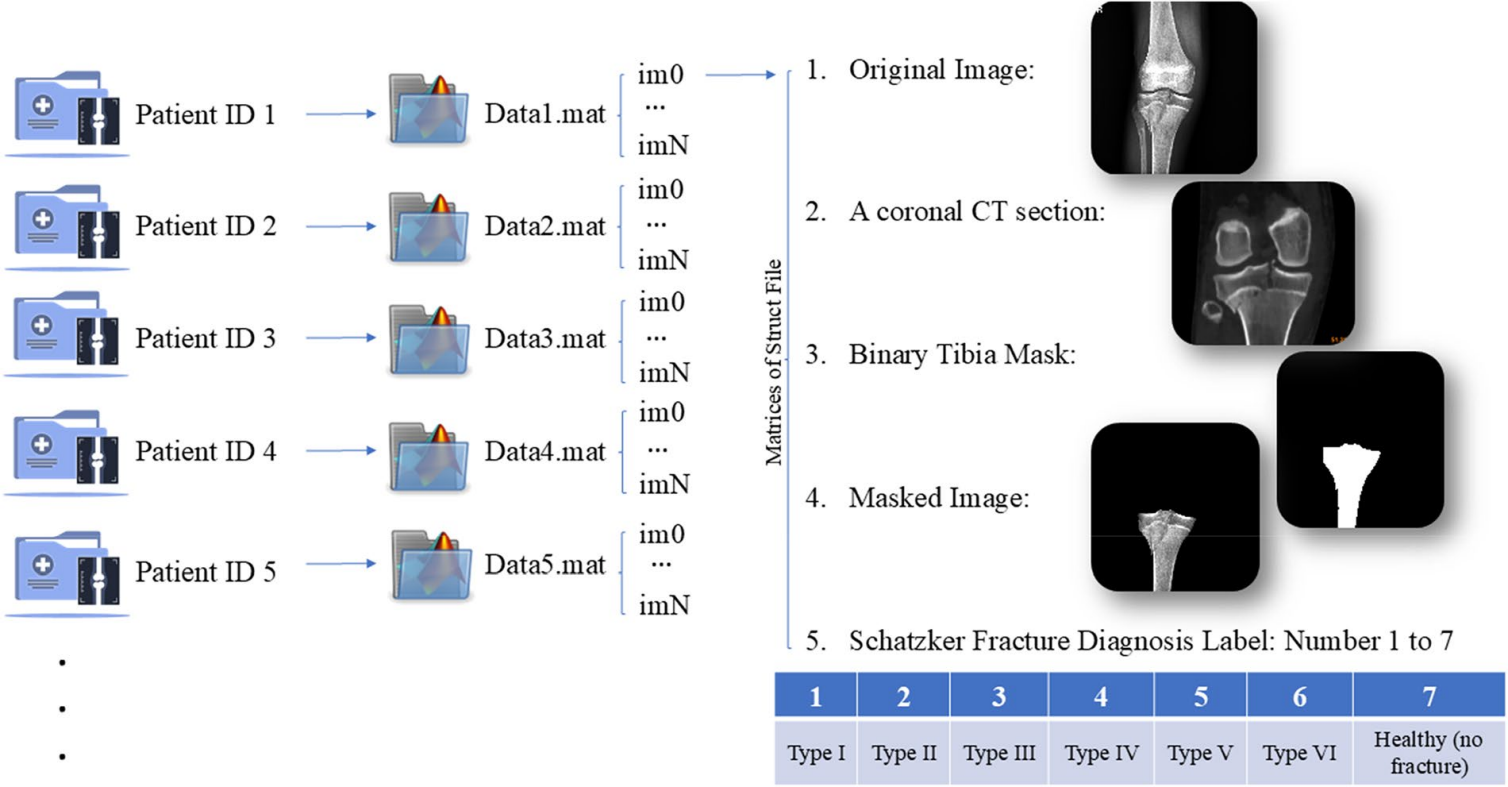
Structured Data Format for Accessing Tibial Plateau Fracture Diagnoses and Patients’ Images.

All data were anonymized and prepared following ethical standards. Researchers are encouraged to use the dataset for academic and non-commercial research purposes. Furthermore, to illustrate the improved accessibility of the proposed data format, Fig. 5 is presented. Patient folders are systematically arranged in sequence and linked to unique patient IDs within this structure. Each folder contains one file with the “.mat” extension. This file may include one or more images (indexed as im0, im1, …, imN) depending on the available imaging data for each patient. A single coronal CT section, common to all images in the file, is included. For each image, five associated data elements are provided: the original radiograph, the shared coronal CT section, a binary mask of the tibia, a segmented tibia image, and the Schatzker classification label. Notably, in cases where bilateral radiographs are available, the left and right legs may exhibit different fracture patterns and thus be assigned distinct Schatzker classification labels.

### Declaration of  AI-assisted technologies in the writing process

During the preparation of this work, the author(s) used ChatGPT (version GPT-4) to enhance the article’s language, grammar, and readability. The authors acknowledge using AI tools solely for language refinement, and these tools did not affect the scientific content, data analysis, or interpretation of results.

## Data Availability

The dataset utilized in this study is publicly available in the cloud and can be accessed on Zenodo (10.5281/zenodo.18007397)^18^ under the Creative Commons Attribution 4.0 International (CC BY 4.0) license. All data were anonymized and prepared following ethical standards. Researchers are encouraged to use the dataset for academic and non-commercial research purposes.

## Code availability

The dataset provides patient-specific data in MATLAB .mat files, each containing structured information for tibial plateau fracture images and segmentation masks. The MATLAB and Python scripts for reading, processing, and visualizing the data are available at the following repository: https://github.com/ali-kazemi8/PlaTiF-Tibial-Plateau-Fracture-Dataset.git.
